# Peritoneal Metastases: Evolution from a Dark Horizon to an Encouraging Present and a Promising Future

**DOI:** 10.3390/jcm12247536

**Published:** 2023-12-06

**Authors:** Manuel Diez-Alonso, Alberto San-Juan, Miguel Angel Ortega, Alberto Gutiérrez-Calvo

**Affiliations:** 1 General Surgery Service, University Hospital Príncipe de Asturias, 28820 Alcalá de Henares, Spain; alberto.gutierrez@salud.madrid.org; 2 Medical Oncology Service, University Hospital Príncipe de Asturias, 28820 Alcalá de Henares, Spain; albsanjuan_2@hotmail.com; 3 Department of Medicine and Medical Specialities, Faculty of Medicine and Health Sciences, University of Alcalá de Henares, 28880 Alcala de Henares, Spain; miguel.angel.ortega92@gmail.com

Peritoneal metastasis (PM) is the primary pattern of metastasis for primary tumours of the appendix, ovary, and peritoneal mesothelioma. There is a modest risk of PM in patients with colorectal or gastric cancer; however, it is much less common in patients with other tumours, such as soft tissue sarcoma, breast cancer, melanoma, and lung cancer [1]. Traditionally, the presence of PM has been considered an incurable situation; after their diagnosis, patients were informed that their prognosis was only a few months and that there was no effective treatment. The survival of patients with PM is poorer than that of patients with metastases in other sites, such as the liver or lungs. Additionally, it is known that the survival of patients with metastasis decreases if the peritoneum is affected. In fact, the eighth actualisation of the American Joint Committee on Cancer’s (AJCC) TNM classification system for Colorectal Cancer, published in 2016, established a new M1 subclass named M1c in order to include patients with PM due to their poor prognosis of survival. However, during recent years, a number of significant therapeutic contributions have opened an expectancy of a promising future so that this condition may no longer be considered a death sentence [1,2,3]. Several factors have contributed to this change. Our knowledge about the biology of peritoneal disease, its clinical behaviour, and the prognostic factors for survival has progressed, and we know that it is not a uniform disease. the introduction of modern chemotherapeutic drugs and biologically targeted agents, with superior cytotoxic efficacy and less toxic effects, have also changed the treatment [1,2,3,4,5]. In addition, cytoreductive surgery (CRS), either accompanied or unaccompanied by hyperthermic intraperitoneal chemotherapy (HIPEC), has been introduced for the treatment of patients with PM with promising results and a great degree of acceptance. Thus, PM can no longer be considered as a process with an inevitable lethal end.

In approximately 25% of cases, PM appears as the sole presentation site of metastatic disease. Most patients diagnosed with PM undergo systemic treatment with chemotherapy; however, CRS with or without HIPEC is a reasonable option in patients with low-risk comorbidities and a low-to-moderate extent of peritoneal dissemination [6]. However, CRS/HIPEC possesses high morbidity and mortality rates, and the careful selection of candidates is mandatory.

According to Lambert in a recent review, the first clinical communication of the application of CRS/HIPEC was made by Spratt in 1980 [1]. This author described the technique used for the treatment of a case of PM originating from an appendiceal mucinous neoplasm and described the concept of “peritoneal surface malignancy” as well as the basis for the treatment of PM as a regional disease. EVOCAPE [7] was the first clinical study indicating that some patients with PM could obtain benefit if they are treated with CRS/HIPEC. In that study, when CRS/HIPEC plus multimodal therapy (surgery and best systemic chemotherapy) was used for colorectal, appendiceal, or ovarian cancers, the median survival improved; conversely, similar results could not be reproduced for primary tumours of the pancreas, hepatobiliary organs, and other foregut organs. This trial effectively promoted a new and contemporary surgical approach for patients with resectable PM. However, the technique spread and became generalised thanks to the works and publications of Sugarbaker [6].

CRS/HIPEC has been associated with controversy for years, and its implementation has not been easy. The small number of randomised clinical trials, the difficulty and degree of skill required by the surgical technique, the morbidity associated with the procedure, the lack of homogeneity in its application (open vs. closed HIPEC; HIPEC vs. PIPAC; the selection of perfused chemotherapy), and the lack of knowledge of the contribution of each of the two components (cytoreduction vs. chemotherapy) have contributed to persistent doubts about its efficacy. However, CRS/HIPEC is currently fully accepted, and it is implemented in today’s daily clinical practice throughout the world. Given the limited amount of solid evidence derived from scientifically proven data, the rationale that justifies the use of CRS/HIPEC is based, for the most part, on retrospective and uncontrolled studies. Currently, CRS/HIPEC is the first option of treatment in patients with low-grade mucinous tumours of the appendix, in advanced ovarian tumours, and in patients with colorectal cancer or peritoneal mesothelioma with a low tumour burden (PCI < 20).

The first randomised trial designed to analyse the role of CRS in patients with PM from Colorectal Cancer was conducted in the Netherlands between 1990 and 1998 [8]. It compared 5 FU/palliative surgery versus 5 FU/CRS/HIPEC plus mitomycin C for 90 min at 40.5 °C. Although the size of the analysed sample was very small (105 patients), the obtained results indicated that survival was better in the CRS/HIPEC arm (22.4 months versus 12.6 months) (*p* = 0.032). A French group proposed and reported good results with the use of HIPEC with a perfusion of oxaliplatin for 30 min [9]. This regimen was recently tested in the PRODIGE 7 trial [10], in which the median overall survival was similar in the CRS/HIPEC group (41·7 months) and in the CRS-on ly group (41·2 months). However, this study reported the longest survival in patients with CRC-PM survival at 42 months, demonstrating the benefit of combining the best systemic therapy with CRS. The currently ongoing CAIRO6 trial [11] is exploring the therapeutic benefit of combining systemic perioperative chemotherapy with CRS/HIPEC.

Two lines of research have explored the efficacy of using CRS/HIPEC for the treatment of PM in an initial or subclinical phase. HIPECT4 [12] explored the use of CRS/HIPEC for 60 min with mitomycin C in a prophylactic setting in patients staged as cT4/N0-2/M0 at diagnosis. The conclusions and relevance obtained were that the addition of HIPEC to CRS improved the 3-year locoregional control rate compared with surgery alone. COLOPEC2 [13] analysed the use of an exploratory laparoscopy during the follow-up in pT4 colon cancer patients for the early detection of peritoneal metastases, at 1 month and at 18 months, compared with computed tomographic imaging. This study was designed to detect occult peritoneal disease that was not identified in the COLOPEC [14] and PROPHYOCHIP trials [15].

The OVHIPEC randomised trial evaluated the efficacy of HIPEC in chemotherapy-naive cases of advanced ovarian cancer [16]. Patients received three cycles of neoadjuvant carboplatin/paclitaxel and were randomised to receive either CRS alone or CRS/HIPEC with a perfusion of cisplatin during 90 min of HIPEC, followed by adjuvant chemotherapy. Better progression-free survival and overall survival were observed in the CRS/HIPEC arm.

Not only have surgical procedures evolved [17], but chemotherapy and systemic agents have also contributed to improve the prognoses of PM patients. There are many different novel treatments that have been introduced in recent years and have been approved for the metastases of different tumours, such as antiangiogenic agents, monoclonal antibodies, and biologic or immune modulator drugs or targeted treatments, that have clearly contributed to better response rates, progression-free survival, and overall survival.

Bevacizumab and Aflibercept are two antibodies that act against the Vascular Endothelial Growth Factor that, when combined with chemotherapy, prolong overall survival beyond 24 months in colorectal cancer patients, independent of their RAS statuses. Cetuximab and Panitumumab are monoclonal antibodies that act against the Epidermal Growth Factor Receptor on the cell membrane in tumours with a non-mutated KRAS gene, which blocks the growth of tumour cells. It is known that these types of drugs reduce the progression of various types of tumours, such as colorectal cancer. In addition, the drugs regorafenib and TAS-112 have emerged as third-line or beyond treatments for colorectal tumours and provide improved overall survival.

The incidence of tumours that express microsatellite instability is very low, but this small group of patients could be treated with immunotherapy, with high response rates, survival, and quality of life, without many of the potential severe adverse effects that are associated with chemotherapy treatment. The TOGA trial [18] revealed that HER-2-positive advanced gastric tumours could benefit from the addition of trastuzumab to 5-FU/platinum-based agents. The median overall survival of patients receiving trastuzumab plus chemotherapy was higher than that observed in patients treated with chemotherapy alone (13.8 vs. 11.1 months). ATTRACTION-4 [19] was a randomised, multicentre, double-blind trial that explored the combination of nivolumab with oxaliplatin-based chemotherapy, and it observed significantly improved progression-free survival.

In the treatment of advanced ovarian cancer, there has been critical innovation with the introduction of PARP inhibitors (olaparib, niraparib, and veliparib) as maintenance therapy for stage III/IV after CRS. PARP is an enzyme involved in the repair of damaged DNA that has been identified as a target in cancers that have BRCA1/BRCA2 mutations and/or recombination repair deficiency [20,21].

These different options in the treatment of PM have become an encouraging challenge for medical oncologists; they should choose the best treatment strategy early in the course of the disease and consider the preferred regimens for each stage of the disease to provide patients with the longest survival and best quality of life possible. The clinical management of PM still entails many difficulties, and depending on the type of primary tumour, different therapeutic approaches are available.

There has been a revolution in the treatment of patients with PM as new and different avenues of research have been opened and progress has been made. What could be the roles of laparoscopy and robotic surgery in the future? How can the new modalities of magnetic resonance imaging contribute to the earlier detection and staging of PM? Future developments in molecular profiling methods will allow for the identification of precise molecular changes that are responsible for PM. Advances in immunotherapy, where immune checkpoint inhibitors and other immunotherapeutic strategies are now being studied, have made it a promising option. Drug delivery systems based on nanotechnology have the ability to deliver chemotherapeutic drugs to tumour cells within the peritoneal cavity in a targeted and regulated manner. This strategy could reduce systemic toxicity while increasing the effectiveness of chemotherapy. Another promising strategy to identify the response to therapy and for the early detection of recurrence is the identification of circulating tumour cells. In the future, we will know if it could be a useful tool to improve survival.
